# Medical Degrees and Other Qualifications

**Published:** 1899-09-02

**Authors:** 


### Medical Degrees and Other Qualifications.

In most foreign countries the State concerns itself much more directly than is the case in England with the regulation of medical practice, and in many, although universities may give degrees in medicine, the only recognised means of qualification is the passing of a State examination. In the United Kingdom, however, a very anomalous condition of affairs gradually grew up in times past, according to which many bodies—universities, colleges, trading societies, and even the Archbishop of Canterbury granted degrees or diplomas which gave the right to practise the various branches of the art and science of medicine.

By the Medical Acts passed in the year 1858, and amended in various details by subsequent Acts, the trade in diplomas was brought under more strict regulation, and a General Medical Council was appointed, whose duty it was to institute a register of all properly qualified persons. For this purpose the Council was authorised to issue regulations as to medical education, to visit and inspect the examinations held by the qualifying bodies, to refuse admission to the register to all who had not undergone the prescribed course of education and examination, and finally to expunge from the Register the names of such persons as might be found guilty of infamous conduct in a professional respect Thus it will be understood that the General Medical Council is in no sense to be regarded as having been appointed for the protection or representation of the medical profession, but rather as having been instituted solely for the purpose of enabling the public to ascertain who are qualified medical practitioners and who are not.

With this object in view the General Medical Council has endeavoured in various ways to enforce a minimum standard of professional knowledge, and one of the most important steps taken in this direction has been the abolition of what were called single qualifications. In old days a man might be qualified in surgery but be at the same time a rank outsider in regard to medicine, while he might be a physician and yet be on the level with the quacks in all that pertained to surgery. The Medical Council, however, has simplified all this by refusing to admit anyone to the Register unless he can show evidence of qualification in all three branches of the art—medicine, surgery, and midwifery. Hence various amalgamations between the Colleges of Physicians and of Surgeons with the object of giving complete registerable qualifications; thus we have one Conjoint Examining Board in England, another in Scotland, and another in Ireland. The Society of Apothecaries in England at the same time obtained power to appoint extra examiners in surgery, so that the L.S.A. also became a triple qualification, and arrangements were made by the Apothecaries' Society in Ireland for the same purpose, while the Universities arranged for one course of study and one series of examinations to cover the whole ground. As things stand at present, then, the following are the examining bodies and the degrees and diplomas given by them which admit to the Register. It must be understood, however, that the Universities grant many other degrees and the Colleges many other diplomas. These, however, are of the nature of higher qualifications, and to obtain them the lower ones must first be obtained. The following are all that are of interest to the commencing student:—

### UNIVERSITIES.

### The University of Oxford.

The degrees of M.B. and B.Ch., which, however, can only be obtained after taking a degree in arts.

### The University of Cambridge.

The degrees of M.B. and B.C. These may be taken without obtaining a degree in arts.

Information may be obtained on application to the Registrar of the University, the Registry, Cambridge.

In both the above universities residence is required, and thus the fees for the examination can hardly be considered apart from the other necessary expenses.

### The University of London.

The degree of M.B., which is of itself a complete qualification. The fees come to £15, in addition to which there is a fee of £2 for matriculation, which, however, takes the place of the necessary preliminary examination in arts.

Information may be obtained on application to the Registrar of the University of London, Burlington Gardens, London, W.

### The University of Durham.

The degree of M.B., which is open to women as well as men. One year at least of the necessary five years' course must be spent in attendance at the College of Medicine, Newcastle-upon-Tyne, and it is essential that the preliminary arts examination for degrees should be passed. Including the fee for the latter the total fees for the qualifying degree amount to £26.

Information may be obtained on application to the secretary of the University of Durham Medical College, Newcastle-on-Tyne, or see the Calendar.

### The Victoria University.

The degrees of M.B., Ch.B. Two years at least out of the necessary five years' course must be spent at one or other of the colleges of the University, namely, Owens College, Manchester; University College, Liverpool; or the Yorkshire College, Leeds. An entrance examination must be passed. Including the fees for this, the total fees for the qualifying degree amount to £17.

Information may be obtained on application to the Registrar or the Dean of either of the colleges as above, or from the Calendar.

### The University of Edinburgh.

The degrees of M.B., Ch.B. Two of the five years' course must be taken in Edinburgh. Women students are recognised if the regulations laid down for the conduct of their classes are conformed with. Including the fee for the preliminary examination, which is half a guinea, the total fees for the qualifying degrees amount to 22½ guineas. The examinations of several other bodies are accepted as equivalent to the preliminary.

Information may be obtained by application to the Dean of the Faculty of Medicine, University of Edinburgh; or from the Calendar.

### The University of Glasgow.

The degrees of M.B., Ch.B. Residence for two years is required as at Edinburgh. The total fees for qualification are the same as at Edinburgh—namely, 22½ guineas; but Glasgow is more liberal in regard to re-examination in case of the candidate being rejected, one re-examination being allowed free. On the other hand, for any further re-examination beyond the first the fees are higher than at Edinburgh. Women students are recognised, and the Lady Margaret College is connected with the University.

For further information see Calendar.

### The University of Aberdeen.

The degrees of M.B., Ch.B. Two years of the necessary five years' course must be passed in Aberdeen. The total fees for the qualifying degrees amount to 22½ guineas, including the entrance examination.

For further information see Calendar.

### The University of St. Andrews.

Part of the course may be taken out at St. Andrews and the rest at the University College, Dundee. The fees for the qualifying degrees (M.B., Ch.B.) are the same as at Edinburgh.

Information may be obtained on application to the Dean of the Medical Faculty, University College, Dundee; or see Calendar.

### The University of Dublin.

The degrees of M.B., B.Ch., and B.A.O. These degrees are only conferred on candidates who are graduates in arts, but the arts course may be carried on concurrently with the medical course. The fees for the qualifying medical degrees amount to £17, with 5s. more for matriculation, but the fees for the arts degree must also be taken account of.

Residence is essential.

Information may be obtained on application to the Registrar of the School of Physic in the University of Dublin.

### The Royal University of Ireland.

The degrees of M.B., B.Ch. These are open to both sexes. The total fees, including matriculation, &c., for the qualifying degrees amount to £17.

---

### COLLEGES.

### The Conjoint Examining Board in England.

This Board is formed by the Royal College of Physicians of London and the Royal College of Surgeons of England, and on passing its examinations the candidate obtains a diploma from each body, and thus becomes L.R.C.P., M.R.C.S.

The total fees for qualification amount to 35 guineas (£36 15s.), in addition to the fees for the preliminary examination in arts.

Information may be obtained from the Secretary, Examination Hall, Victoria Embankment, London, W.C.

The Scottish Colleges.

The Royal College of Physicians of Edinburgh, the Royal College of Surgeons of Edinburgh, and the Faculty of Physicians and Surgeons of Glasgow have instituted a system of conjoint examinations, on passing which the candidate obtains the diplomas of each body, and becomes L.R.C.P.Edin., L.R.C.S.Edin., and L.F.P.S.Glasg.

The total fees for this qualification amount to £31, including the preliminary.

Information may be obtained on application to Jas. Robertson, Esq., 48, George Square, Edinburgh; or, in regard to the preliminary examination, to Alexander Mackay, Esq., 40, Princes Street, Edinburgh.

The Conjoint Examining Board in Ireland.

The Royal College of Physicians of Ireland and the Royal College of Surgeons in Ireland have arranged a series of examinations, on passing which the candidate obtains the diploma of each body, and becomes L.R.C.P.I., L.R.C.S.I.

The total fees for this qualification amount to 42 guineas (£44 2s.), including the fee for the preliminary examination.

Information may be obtained from the Registrars of the Colleges.

### THE APOTHECARIES' HALLS.

The Society of Apothecaries of London.

This body grants a full qualification, the L.S.A., the total fees for which amount to 15 guineas (£15 15s.), in addition to the fee for the preliminary examination in arts.

Information may be obtained on application to the Secretary to the Examiners, Apothecaries' Hall, Blackfriars, London, E.C.

The Apothecaries' Hall of Ireland is entitled to grant a full qualification, the fees for which amount to 21 guineas.

Information may be obtained from the Secretary of the Apothecaries' Hall, Dublin.

